# Supplementary material for: Spatial Variation in Stream-Water Bacterial Communities Across Sites Differing in Wild Chinese Giant Salamander Detection Frequency
Source: Biology (Basel). 2026 Sep 18;15(18):1647. doi: 10.3390/biology15181647 (PMC13604818; doi:10.3390/biology15181647)
Supplement: Supplementary file 1 [file biology-15-01647-s001.zip › Data Related to “Spatial Variation in Stream-Water Bacterial Communities across Sites Differing in Wild Chinese Giant Salamander Detection Frequency”/Microbial Data/report/src/hyperlinks/QC_methods_CH.pdf]

中文版Method

仅供客户在文章写作时参考，分析内容和方法请以结题报告为准，请客户自行承担文章查重等相关风险。

1 实验流程

1.1 样本检测

详见样本检测报告。

1.2 PCR产物获取

引物对应区域:

16SV4区引物（515F和806R）：鉴定细菌多样性； 18SV4区引物（528F和706R）：鉴定真核微生物多样性； ITS1区引物（ITS5-1737F和ITS2-2043R）：鉴定真菌多样性。此外，扩增区域还包括 16SV3-V4/16SV4-V5/16SV5-V7；古菌16SV4-V5/古菌 16SV8；18SV9 和ITS2 区。

所有 PCR 混合液加入 15µL Phusion⑥ High-Fidelity PCR Master Mix（New England Biolabs）、0.2µM 引物和10ng 基因组DNA模板， 在98℃下进行1分钟的第一次变性，然后在 98℃（10s）、50℃（30s）和72℃（30s）下进行30次循环，最后在72℃下保持5分钟。

1.3 文库构建及上机测序

DNA样品扩增检测合格后， 进行PCR产物的混合和纯化， 再经末端修复、加A尾、加测序接头、纯化等步骤完成整个文库制备工作。库检合格后， 把不同文库按照有效浓度及目标下机数据量的需求pooling后进行双末端测序。

2 生物信息分析

2.1 数据质控

2.1.1 数据拆分：根据Barcode序列和PCR扩增引物序列从下机数据中拆分出各样本数据。

2.1.2 双端数据拼接：截去Barcode和引物序列后使用FLASH (http://ccb.jhu.edu/software/FLASH/)）（Magoc T et al.,2011），对每个样本的reads进行拼接，得到的拼接序列为原始Tags数据（Raw Tags）。

2.1.3 数据质控：使用fastp软件对拼接得到的Raw Tags经过严格的过滤处理得到高质量的Tags数据（Clean Tags）（Bokulich NAetal.,2012）。

2.1.4 去除嵌合体：经过以上处理后得到的Tags需要进行去除嵌合体序列的处理，Tags序列通过与物种注释数据库（Silva database https://www.arb-silva.de/ for 16S/18S, Unite database https://unite.ut.ee/ for ITS) 进行比对检测嵌合体序列，并最终去除其中的嵌合体序列，得到最终的有效数据（Effective Tags）（Edgar RC etal.,2011）。

客户引物项目默认均与silva注释数据库（https://www.arb-silva.de/ ）进行比对检测嵌合体序列并去除。

若客户提供双端原始数据（raw data），将根据客户指定的扩增区域，选择对应数据库（Silva database, https://www.arb-silva.de/ for 16S/18S, Unite database, https://unite.ut.ee/ for ITS）进行嵌合体去除；若客户提供单端数据，是否去除嵌合体将根据数据类型决定：如为已质控的 clean，则不进行嵌合体去除；如为未质控的，则根据扩增区域选择对应数据库去除嵌合体。

3 Reference

Bokulich NA, SubramanianS, Faith JJ, et al. Quality-filtering vastly improves diversity estimates from Illumina amplicon sequencing.Nature Methods. 2012;10(1):57-59. doi:10.1038/nmeth.2276.

Edgar RC, Haas BJ, Clemente JC, Quince C, Knight R. UCHIME improves sensitivity and speed of chimera detection.Bioinformatics. 2011;27(16):2194-2200. doi:10.1093/bioinformatics/btr381.

MagocT, Salzberg SL. FLASH: fast length adjustment of short reads to improve genome assemblies.Bioinformatics. 2011;27(21):2957-2963. doi:10.1093/bioinformatics/btr507.
